# 晚期NSCLC患者血清*EGFR*基因突变状态的测定及意义

**DOI:** 10.3779/j.issn.1009-3419.2013.06.06

**Published:** 2013-06-20

**Authors:** 玲 马, 莉 刘, 涛 张, 莉 单

**Affiliations:** 830011 新疆，新疆医科大学附属肿瘤医院肺内一科 Department of Medical Oncology, Tumor Hospital Affiliated to Xinjiang Medical University, Urumqi 830011, China

**Keywords:** 肺肿瘤, 血清, 表皮生长因子受体, 酪氨酸激酶抑制剂, 疗效, Lung neoplasms, Serum, Epidermal growth factor receptor, Tyrosine kinase inhibitor, Efficacy

## Abstract

**背景与目的:**

小分子酪氨酸激酶抑制剂（tyrosine kinase inhibitors, TKIs）对于表皮生长因子受体（epidermal growth factor receptor, *EGFR*）基因突变的肺癌患者显示出良好的治疗效果。本研究旨在探讨晚期非小细胞肺癌（non-small cell lung cancer, NSCLC）患者血清*EGFR*基因突变状态与EGFR-TKIs疗效的关系。

**方法:**

检测80例一线口服EGFR-TKIs晚期NSCLC患者血清*EGFR*基因的突变状态，对患者进行长期随访并评价治疗效果。

**结果:**

80例患者血清*EGFR*基因突变27例（33.8%），其中外显子19缺失突变12例（44.4%），外显子21点突变15例（55.6%）；血清*EGFR*基因突变患者的有效率（55.6%, 15/27）高于野生型患者（17.0%, 9/53），差异具有统计学意义（*χ*^2^=0.370, *P* < 0.001）；血清*EGFR*基因突变患者中位无进展生存时间（progress free survival, PFS）明显长于野生型患者（9.8个月*vs* 5.7个月，*P*=0.014）。

**结论:**

血清*EGFR*基因突变患者一线口服EGFR-TKIs的疗效优于野生型患者，血清*EGFR*基因状态可为EGFR-TKIs的一线治疗提供有效依据。

## Abstract (en)

**Background and objective:**

Epidermal growth factor receptor tyrosine kinase inhibitors (EGFR-TKIs) has shown a high response rate in the treatment of lung cancer in patients with (*EGFR*) mutation. The aim of this study is to evaluate the relationship between *EGFR* mutation status in serum and predicting benefit from EGFR-TKIs therapy in patients with advanced non-small cell lung cancer (NSCLC).

**Methods:**

We examined *EGFR* mutation status in serum of 80 patients with advanced, EGFR-TKIs given as first-line therapy NSCLC. All patients were received long-term follow-up, and the drug efficacy were observed and evaluated.

**Results:**

The *EGFR* mutation in serum was detected in 33.8% (27/80) of NSCLC patients examined, in which exon 19 deletion mutation was present at a frequency of 44.4% (12/27) and exon 21 point mutation was 55.6% (15/27); The response rate to EGFR-TKI in patients with *EGFR* mutation in serum was (55.6%, 15/27), which was remarkably higher than that in *EGFR* wild-type patients (17.0%, 9/53), the difference was statistically significant (*χ*^2^=0.370, *P* < 0.001); The median progression free survival (PFS) of patients with *EGFR* mutation in serum was remarkably better than that of *EGFR* wild-type patients (9.8 months *vs* 5.7 months, *P*=0.014).

**Conclusion:**

In patients with advanced, EGFR-positive in serum NSCLC, EGFR-TKIs given as first-line therapy is associated with improved drug efficacy. The results suggest that it is feasible to use serum to detect *EGFR* mutation, which can predict a benefit from EGFR-TKIs given as first-line therapy.

肺癌是最常见的恶性肿瘤之一，其发病率与死亡率已跃居首位，约80%的患者为非小细胞肺癌（non-small cell lung cancer, NSCLC）^[1]^，大多数患者在发现时已属晚期，中位生存时间约8个-10个月，以铂类为基础的化疗是这部分患者的主要治疗手段，但疗效似乎已达到平台期。近年来，小分子酪氨酸激酶抑制剂（tyrosine kinase inhibitors, TKIs）等靶向药物在NSCLC治疗中的地位日益突出，尤其是表皮生长因子受体（epidermal growth factor receptor, *EGFR*）基因突变的患者，治疗效果不亚于化疗，且毒副反应较轻^[2]^。肺原发肿瘤组织是检测*EGFR*基因突变状况的主要标本来源，可由气管镜或肿物穿刺获取，但是部分患者由于各种原因并不能获取肿瘤组织标本，无法进一步检测*EGFR*基因突变状况为EGFR-TKIs治疗提供有效依据。本研究旨在探讨晚期NSCLC患者血清*EGFR*基因突变状况与EGFR-TKIs疗效的相关性。

## 1 资料与方法

### 1.1 研究对象

以2010年12月-2011年12月就诊于我院一线口服EGFR-TKIs治疗的80例晚期NSCLC患者为研究对象，其中男性38例，女性42例，年龄为35岁-78岁，平均（63.2±5.6）岁；均经组织学或细胞学确诊为NSCLC，且行血清*EGFR*基因突变检测；经pTNM分期为Ⅲb期-Ⅳ期，丧失手术或放疗根治时机；所有患者为拒绝化疗或体能状况差不能接受化疗而选择EGFR-TKIs作为一线治疗，其中因患方拒绝化疗并要求靶向治疗者为21例，因PS评分差无法耐受化疗而接受靶向治疗者有32例，根据NCCN指南推荐患者进行靶向治疗者有27例；口服吉非替尼患者53例，厄洛替尼27例；治疗前检查无明显肺间质性病变，血常规及心、肝、肾等重要脏器功能基本正常；所有患者均签署治疗知情及标本留取同意书。

### 1.2 标本收集及基因检测

使用带有血清分离胶的血清管，抽取患者空腹外周静脉血4 mL，静置于常温下，血清管离心3, 000 rpm、5 min，分离上清液。使用德国Qiagen生产的血液DNA提取试剂盒提取DNA，无菌蒸馏水对所提取的DNA进行反复洗脱，分光光度法测定DNA的浓度和纯度，-20 ℃存储所提取的DNA；采用扩增受阻突变系统（amplification refractory mutation system）方法进行PCR扩增，检测*EGFR*基因外显子19缺失突变和/或外显子21点突变。用厦门艾德生物医药科技有限公司*EGFR*基因突变检测试剂盒（人类*EGFR*基因21种突变检测试剂盒）进行检测，实验具体操作步骤参照试剂盒说明书进行。采用StrataGene MX3000P实时PCR仪进行扩增，每次检测样品包括1个阳性质控品、1个NTC对照。如果Ct值为0或Ct值> 30，则实验结果判为野生型。所述荧光PCR的反应条件：95 ℃预变性5 min，1个循环；95 ℃变性25 s，64 ℃退火20 s，72 ℃延伸20 s，15个循环；93 ℃变性25 s，60 ℃退火35 s，72 ℃延伸20 s，31个循环。

### 1.3 结局指标及随访

每位患者从用药开始详细记录症状缓解的时间，出现不良反应的时间、程度及耐受情况；所有患者于口服2个月后进行首次评价，行胸片、CT、肿瘤标记物、肝肾功及远处转移部位的客观检查，以后每1个月住院复查或门诊、电话随访。根据实体肿瘤疗效评价标准对病灶进行疗效评价，分为完全缓解（complete response, CR）、部分缓解（partial response, PR）、疾病稳定（stable disease, SD）和疾病进展（progressive disease, PD），有效率（response rate, RR）=（CR+PR）/总例数×100%，临床获益率（clinical benefit rate, CBR）=（CR+PR+SD）/总例数×100%；随访患者无进展生存时间（progression free survival, PFS），PFS是指肿瘤患者从接受靶向治疗开始，到观察到疾病进展或者发生因为任何原因的死亡之间的这段时间；本组研究80例患者，失访2例，失访率为2.5%。

### 1.4 统计学方法

数据采用SPSS 17.0统计软件包进行处理，定性数据以百分比进行表示，计数资料采用*χ*^2^检验，*Kaplan-Meier*法绘制生存曲线，当*P* < 0.05时差异有统计学意义。

## 2 结果

### 2.1 血清*EGFR*基因突变与临床特征的关系

80例患者共检测到血清*EGFR*基因突变27例（33.8%），其中女性（47.1%, 16/34）高于男性患者（23.9%, 11/46），腺癌（41.4%, 24/58）高于非腺癌患者（13.6%, 3/22），非吸烟患者（46.9%, 15/32）高于吸烟患者（25.0%, 12/48），差异具有统计学意义（*P* < 0.05）；27例*EGFR*基因突变的患者中，外显子19缺失突变12例（44.4%），而外显子21点突变15例（55.6%）；基因突变状态与年龄、临床分期、PS评分无关（*P* > 0.05）（表 1）。

**1:** 血清*EGFR*基因突变与患者临床特征关系[*n*(%)] The relationship of *EGFR* mutation statuses in serum and clinical characteristics [*n*(%)]

Characteristic	*n*	*EGFR* mutation status in serum		*χ*^2^	*P*
		Positive (*n*=27)	Negative (*n*=53)		
Age (year)				1.392	0.238
< 65	43	17 (39.5)	26 (60.5)		
≥65	37	10 (27.0)	27 (73.0)		
Gender				4.684	0.030
Male	46	11(23.9)	35 (76.1)		
Female	34	16 (47.1)	18 (52.9)		
Pathology				5.490	0.019
Adenocarcinoma	58	24 (41.4)	34 (58.6)		
Non-adenocarcinoma	22	3 (13.6)	19 (86.4)		
Smoking history				4.109	0.043
Ever	48	12 (25.0)	36 (75.0)		
Never	32	15 (46.9)	17 (53.1)		
TNM				0.414	0.520
Ⅲb	15	4 (26.7)	11 (73.3)		
Ⅳ	65	23 (35.4)	42 (64.6)		
ECOG PS				0.747	0.388
0-1	38	11 (28.9)	27 (71.1)		
≥2	42	16 (38.1)	26 (61.9)		

### 2.2 血清*EGFR*基因突变与近期疗效的关系

血清*EGFR*基因突变型患者的RR（55.6%, 15/27）高于野生型患者（17.0%, 9/53），差异具有统计学意义（*χ*^2^=0.370，*P* < 0.001）；*EGFR*基因突变型患者的CBR（92.6%, 25/27）虽高于野生型患者（86.8%, 46/53），但差异无统计学意义（*χ*^2^=0.086, *P*=0.438）（表 2）。

**2:** 血清*EGFR*基因突变与近期疗效的关系[*n* (%)] The relationship of *EGFR* mutation statuses in serum and efficacy [*n* (%)]

Efficacy	*EGFR* mutation sratus in serum	
	Positive (*n*=27)	Negative (*n*=53)
CR	1 (3.7)	0 (0)
PR	14 (51.9)	9 (17.0)
SD	10 (37.0)	37 (69.8)
PD	2 (7.4)	7 (13.2)
*χ*^2^=0.378, *P*=0.004. CR: complete response; PR: partial response; SD: stable disease; PD: progressive disease.		

### 2.3 血清*EGFR*基因突变与PFS的关系

末次随访时间为2013年1月，中位随访时间为7.8（3.5-26）个月；血清*EGFR*基因突变型患者中位PFS明显长于野生型患者（9.8个月*vs* 5.7个月，*P*=0.014）（图 1）；进一步对突变患者进行分层分析，外显子19缺失患者的中位PFS优于外显子21点突变的患者，但差异无统计学意义（11.2个月*vs* 8.8个月，*P*=0.328）（图 2）。

**1:**
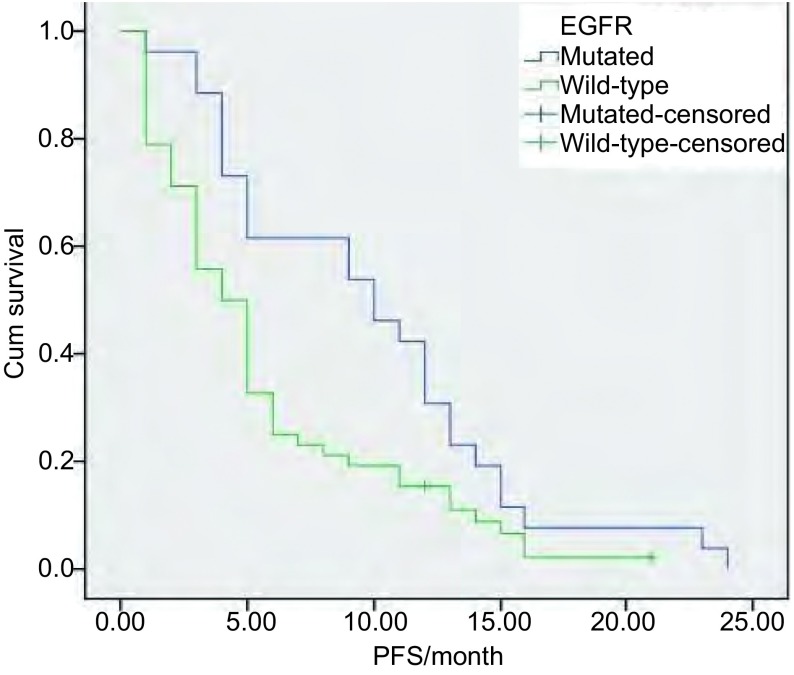
血清*EGFR*基因突变型与野生型患者的PFS曲线 The PFS surve of patients with serum *EGFR* gene mutation and wild-type gene. PFS: progression free survival.

**2:**
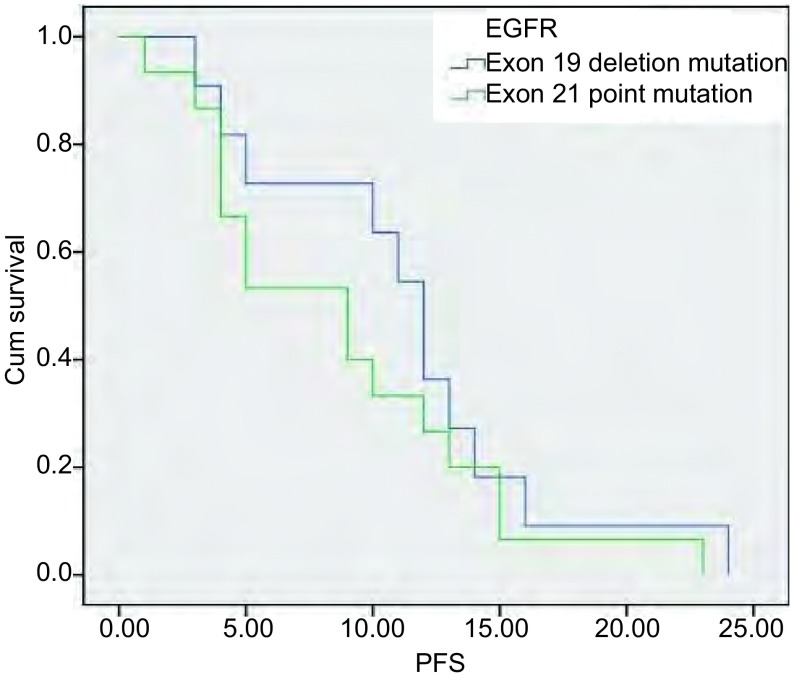
血清*EGFR*基因外显子19与21突变患者的PFS曲线 The PFS curves of patients with serum *EGFR* exon 19 and 21 mutations

## 3 讨论

检测*EGFR*基因突变状态并预测晚期NSCLC患者口服EGFR-TKIs的疗效已成为定论，*EGFR*基因突变型的患者的有效率明显高于野生型患者，且其治疗效果不亚于含铂的两药联合化疗方案^[3-5]^。*EGFR*基因位于人类7号染色体的短臂上，由188, 307个碱基组成，包括28个外显子，主要是在外显子18-21上会发生突变，而这些突变与靶向药物反应性有关，原因可能是这些突变改变了*EGFR*基因胞内ATP结合区的结构，提高了它对靶向药物的结合能力^[6]^。目前，虽然已经报道发现30余种突变与药物反应性有关，但主要是外显子19的缺失突变和外显子21的点突变。相关研究^[7]^显示，*EGFR*基因突变在亚裔NSCLC患者中约占30%-40%，在西方人群中约为10%。一项西班牙研究^[8]^报道，*EGFR*基因突变中，外显子19缺失突变率为62.2%，外显子21点突变率为37.8%。在本研究入组的80例NSCLC患者中，发生外显子19缺失突变为12例，外显子21点突变有15例，外显子19缺失突变率为44.4%（12/27），外显子21点突变率为55.6%（15/27），与文献报道范围略有差异，可能与本组研究样本量较少有关。通过分层分析，二者经EGFR-TKIs治疗后的PFS虽有2.4个月的差别（外显子19缺失突变11.2个月*vs*外显子21点突变8.8个月），但统计学没有明显差异，提示存在这两种突变的NSCLC患者均可从一线EGFR-TKIs治疗中取得较好的疗效。

目前进行基因检测都需要通过有创性组织活检，而且不易重复获取，部分患者病理标本获取率低，限制了靶向治疗的应用，因此寻找肿瘤组织替代方案用于基因检测是当前迫切需要解决的问题。研究^[9-11]^表明，NSCLC患者的胸水、唾液、粪便和血浆中会有少量突变的肿瘤细胞DNA，尤其在患者的外周血中。周小昀等^[10]^通过检测170例NSCLC患者外周血清游离DNA中*EGFR*基因突变情况，发现全组患者中敏感突变率为35%，应用EGFR-TKIs治疗的有效率高达83%。本组研究中，80例晚期NSCLC患者血清EGFR突变率为33.8%，而EGFR-TKIs治疗有效率为55.6%，明显低于周小昀等的研究，考虑可能与该研究中腺癌入组率高、研究对象多为EGFR-TKIs治疗优势人群有关。Pathak等^[12]^报道外周血中肿瘤DNA来源有4种可能：①原发肿瘤释放的DNA；②外周循环中肿瘤微转移灶释放的DNA；③肿瘤细胞的凋亡释放的DNA；④肿瘤细胞的坏死释放的DNA。肿瘤患者外周血游离DNA含量比正常人增高10倍。晚期肺癌患者血清游离DNA中可检测到*EGFR*突变，而文献报道的早期肺癌患者检测到突变较少的原因可能是由于相对于早期肺癌患者，晚期肺癌患者瘤负荷大，转移途径多，部位广，通过血行播散的几率高，因此检测阳性率更高。本研中Ⅲb期与Ⅳ期患者血清*EGFR*基因突变率相差无几，考虑为样本含量较少，掩盖了总体的规律，这尚需大样本的研究进一步探讨。

研究^[13, 14]^报道EGFR-TKIs一线治疗晚期NSCLC有近40%的客观有效率，疾病控制率约70%，PFS达到9个月以上，且患者不良反应轻微，耐受性较好，安全性良好。吴一龙等^[15]^研究表明，*EGFR*基因突变的NSCLC患者，使用EGFR-TKIs治疗的PFS明显高于化疗方案。同样，Sirera等^[16]^研究中*EGFR*敏感突变的患者接受EGFR-TKIs治疗的总体RR，比接受化疗的患者提高2倍-3倍。本研究对80例一线口服EGFR-TKIs治疗的晚期NSCLC患者血清*EGFR*基因突变状况进行了检测，结果发现27例患者血清*EGFR*基因发生了突变，突变率为33.8%，其中女性（47.1%, 16/34）高于男性患者（23.9%, 11/46），腺癌（41.4%, 24/58）高于非腺癌患者（13.6%, 3/22），非吸烟（46.9%, 15/32）高于吸烟患者（25.0%, 12/48），差异具有统计学意义（*P* < 0.05），显示女性、非吸烟及腺癌患者突变率更高，与IPASS研究^[2]^报道的结果相似。本组研究当中，血清*EGFR*基因突变的晚期NSCLC患者使用EGFR-TKIs治疗的RR为55.6%，PFS为9.8个月，而野生型患者的RR为17.0%，PFS为5.7个月，无论是RR还是中位PFS，二者之间均具有统计学差异，印证了检测*EGFR*基因突变对于患者实现个体化治疗具有非常重要的意义。

综上所述，对于组织标本获取困难，后期需要进行靶向治疗的晚期NSCLC患者而言，通过血清标本检测*EGFR*基因突变状况，不失为一种取材方便、痛苦小、简便易行的方法，可能为为临床用药提供依据。
